# Effect of Al Element on the Microstructure and Properties of Cu-Ni-Fe-Mn Alloys

**DOI:** 10.3390/ma11091777

**Published:** 2018-09-19

**Authors:** Ran Yang, Jiuba Wen, Yanjun Zhou, Kexing Song, Zhengcheng Song

**Affiliations:** 1 School of Materials Science and Engineering, Henan University of Science and Technology, Luoyang 471023, China; yr136@163.com (R.Y.); dazhou456@163.com (Y.Z.); 18438583959@163.com (Z.S.); 2 Collaborative Innovation Center of Nonferrous Metals, Henan University of Science and Technology, Luoyang 471023, China

**Keywords:** Cu-Ni-Al alloy, microstructure, mechanical property, corrosion resistance

## Abstract

The effects of aluminum on the mechanical properties and corrosion behavior in artificial seawater of Cu-Ni-Fe-Mn alloys were investigated. Cu-7Ni-*x*Al-1Fe-1Mn samples, consisting of 0, 1, 3, 5, and 7 wt % aluminum along with the same contents of other alloying elements (Ni, Fe, and Mn), were prepared. The microstructure of Cu-7Ni-*x*Al-1Fe-1Mn alloy was analyzed by Transmission Electron Microscopy (TEM), and its corrosion property was tested by an electrochemical system. The results show that the mechanical and corrosion properties of Cu-7Ni-*x*Al-1Fe-1Mn alloy have an obvious change with the aluminum content. The tensile strength has a peak value of 395 MPa by adding 3 wt % aluminum in the alloy. Moreover, the corrosion rate in artificial seawater of Cu-7Ni-3Al-1Fe-1Mn alloy is 0.0215 mm/a which exhibits a better corrosion resistance than the commercially used UNS C70600. It is confirmed that the second-phase transformation of Cu-7Ni-*x*Al-1Fe-1Mn alloy follows the sequence of α solid solution → Ni_3_Al → Ni_3_Al + NiAl → Ni_3_Al + NiAl_3_. The electrochemical impedance spectroscopy (EIS) shows that the adding element aluminum in the Cupronickel can improve the corrosion resistance of Cu-7Ni-*x*Al-1Fe-1Mn alloy.

## 1. Introduction

Due to their high resistance to biofouling, heat transfer ability, and favorable resistance to the ravages of the marine environment, copper-based alloys are being offered as solutions to a range of industries requiring reliability in seawater to meet today’s exacting engineering challenges. It is well known that the addition of nickel to copper matrix can usually improve the comprehensive properties of the alloy, including corrosion and mechanical properties [1,2,3]. For instance, H. Nady et al. [4] investigated the stability of Cu-Al-Ni alloy in neutral chloride solutions with different nickel contents. It is confirmed that the corrosion rate of the alloy decreases as the content of nickel increases. However, its cost is quite high due to its high nickel content. In the 1930s, it was found that adding appropriate amounts of iron and manganese to copper–nickel alloys could improve corrosion–erosion resistance [5,6,7]. The most commonly used for seawater pipelines is the UNS C70600.

The desired properties, including corrosion and mechanical properties, can be achieved by alloying elements aluminum [8,9,10,11]. One of the copper-based alloys Cu-14.5Ni-3Al-1.3Fe-0.3Mn has found increasing use in recent years. In previous studies [12,13,14], the good corrosion resistance of Cu-Ni-Al alloy in chloride solution was found to be attributed to the formation of a duplex oxide film. However, the effect of aluminum on the combination property of special Cupronickel alloys Cu-7Ni-*x*Al-1Fe-1Mn has not been in-depth studied. Thus, a study of aluminum effect of Cu-7Ni-*x*Al-1Fe-1Mn on the mechanical properties and corrosion resistance is necessary.

For copper–nickel alloys, its ability of both mechanical strength and corrosion resistance in seawater is crucial for engineering application. Therefore, we tried to reduce nickel content by adding aluminum and expected better results with the Cupronickel alloy of Cu-7Ni-*x*Al-1Fe-1Mn alloy (wt.%, used throughout the whole paper). We prepared Cu-7Ni-*x*Al-1Fe-1Mn with different aluminum contents and carried out tensile tests and immersion test. The experimental results will provide guidance for the research and industrial application of Cu-7Ni-*x*Al-1Fe-1Mn alloy.

## 2. Experimental

### 2.1. Materials

The nominal compositions of the designed Cu-Ni-Al alloys are listed in Table 1. The raw materials were electrolytic copper, nickel block, electrolytic aluminum, Cu-10 mass% iron master alloy, and Cu-22 mass% manganese master alloy. The raw materials were smelted in medium frequency vacuum induction furnace by feeding sequence of copper, nickel, Cu-Fe master alloy, Cu-Mn master alloy, and electrolytic aluminum. The raw materials were melted in proper ratios at 1170–1200 °C and deoxidized by magnesium alloy.

### 2.2. Experimental Method

**Figure 1:**
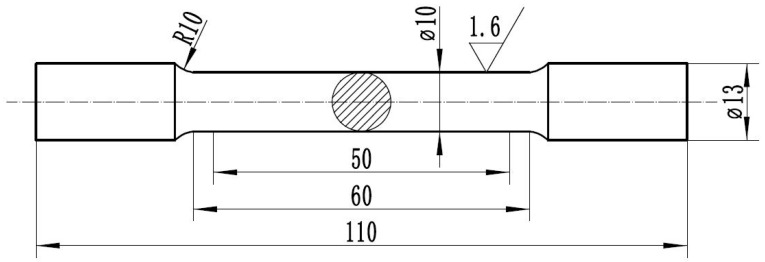
Drawing of tensile specimens (unit: mm).

Mechanical properties of Cu-7Ni-*x*Al-1Fe-1Mn alloy were measured in accordance with the uniaxial tensile test. Tensile samples were prepared according to the GB/T 228.1-2010 method of test at room temperature. Figure 1 shows the tensile specimens for room temperature tests. Tensile testing was performed using a SHIMADZU AG-I250KN precision universal testing machine with a constant strain rate of 1 mm/min. For transmission electron microscopy (TEM), thin foil specimens with a diameter 3 mm were prepared by means of both a double-jet electropolisher and an ion thinner. Microstructures of alloys were observed by JEOL JEM-2100.

The corrosion resistance of Cu-7Ni-*x*Al-1Fe-1Mn alloy was measured by static immersion corrosion test based on JB/T 7901-1999, and the corrosion rate was calculated by weight loss method. The sample size was Φ30 mm × 2.5 mm, and each group had three parallel specimens. The surface area of the sample was measured before immersion corrosion, and the quality was accurately determined by an analytical balance.

The corrosion medium was artificial seawater; the artificial seawater composition is shown in Table 2 [15]. The test was carried out at room temperature for 10 days, and the corrosion medium was replaced every seventh day. After completion of the test, with the volume ratio of 1:2 hydrochloric acid solution, on the corrosion after cleaning, the corrosion products were removed after cleaning with alcohol, and then the sample was put in a drying oven drying to calculate the quality loss of samples before and after corrosion. The corrosion rate *R* was calculated by the change of sample quality before and after corrosion. Then the corrosion rate was calculated according to Equation (1):

(1) $$R=\frac{8.76\times 10^{7}\times (M-M_{1})}{STD}$$

where *R* is the corrosion rate, mm/a; *M* is the quality before metal corrosion, g; *M*_1_ is quality of the sample after removing the corrosion product, g; *S* is the surface area of the sample, m^2^; *T* is the corrosion time, h; and *D* is the density, Kg/m^3^.

Electrochemical tests were conducted in artificial seawater and exposed to laboratory air. The working electrodes were plastic pipes by epoxy resin with a surface area of 1 cm^2^ to contact the solution. A three-electrode system with graphite electrode as auxiliary electrode and saturated calomel as reference electrode was used in the electrochemical experiment. The AC impedance were measured in the frequency range from 0.1 to 10^5^ Hz. The amplitude of the superimposed ac-signal was 10 mV. Each experiment was conducted at least three times to ensure the stability of the test.

## 3. Results and Discussion

### 3.1. Microstructure of As-Cast Alloy

**Figure 2:**
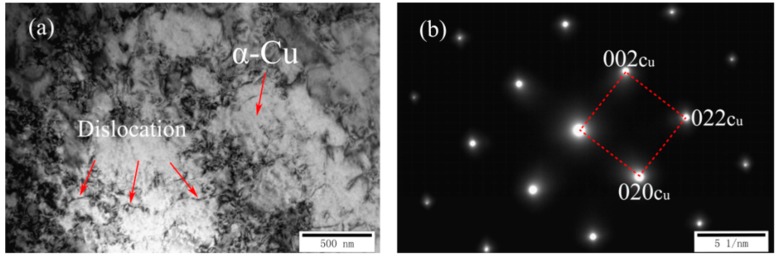
Microstructures of as-cast Cu-7Ni-1Fe-1Mn alloy: (**a**) TEM image; and (**b**) the corresponding SADP of (**a**).

Figure 2a,b shows the TEM and corresponding selected area diffraction patterns (SADP) of as-cast Cu-7Ni-1Fe-1Mn alloy, respectively. Comparing with the standard electron diffraction (SED), the microstructure of the alloy was a α copper matrix with the incident axis of the [100] band, and there was no visible second phase structure.

**Figure 3:**
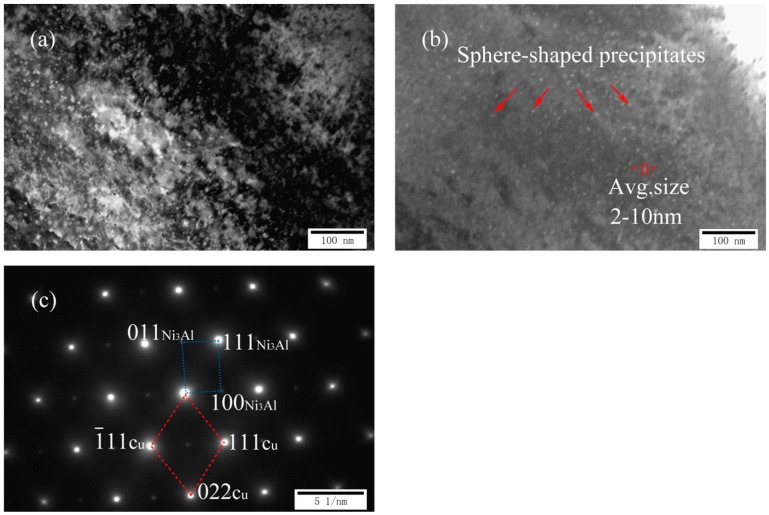
Microstructures of cast Cu-7Ni-lAl-1Fe-1Mn alloy: (**a**) bright-field image; (**b**) dark-field image; and (**c**) the corresponding SADP of (**a**).

Figure 3a,c shows two TEM images and a corresponding SADP of as-cast Cu-7Ni-1Al-1Fe-1Mn alloy, respectively. Compared with cast Cu-7Ni-1Fe-1Mn alloy, a dispersed, small spherical second phase with a diameter of about 2–10 nm began to appear on the copper substrate (Figure 3a,b). According to the analysis of diffraction patterns (Figure 3c), the precipitates is one of the nickel–aluminum intermetallic compounds, namely Ni_3_Al, with a body centered cubic structure. Phase relationship of copper matrix and precipitate is ($\bar{1}$11) Cu//(111)Ni_3_Al, (022) Cu//(011)Ni_3_Al, [0$\bar{1}$1] Cu//[0$\bar{1}$1] Ni_3_Al.

**Figure 4:**
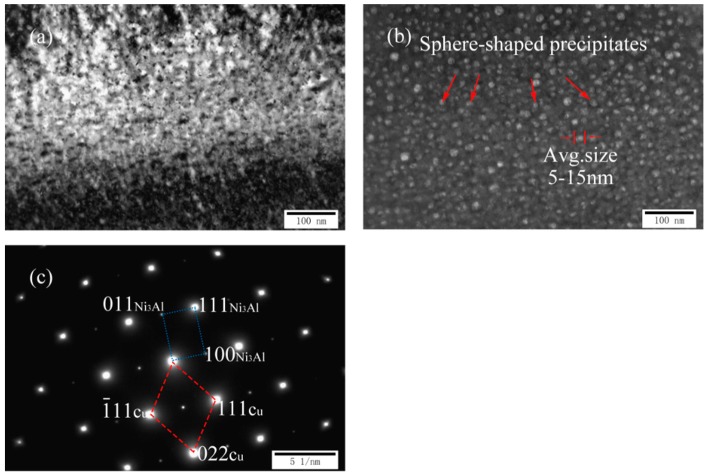
Microstructures of cast Cu-7Ni-3Al-1Fe-1Mn alloy: (**a**) bright-field image; (**b**) dark-field image; and (**c**) the corresponding SADP of (**a**).

Figure 4a–c exhibits the TEM images and corresponding selected area diffraction patterns (SADP) of as-cast Cu-7Ni-3Al-1Fe-1Mn alloy. As can be seen in Figure 4a,b, many second phases having a diameter of about 5–15 nm were distributed over the copper substrate. Compared with the precipitated structure of the cast Cu-7Ni-1Al-1Fe-1Mn alloy, the morphology of the second phase did not change, and the size increased from 2–10 to 5–15 nm. As shown in Figure 4c, the structure of the precipitate did not change, the orientation relationship between precipitate and the surrounding Cu-matrix is found as follows: ($\bar{1}$11) Cu//(111)Ni_3_Al, (022) Cu//(011)Ni_3_Al, [0$\bar{1}$1] Cu//[0$\bar{1}$1] Ni_3_Al.

**Figure 5:**
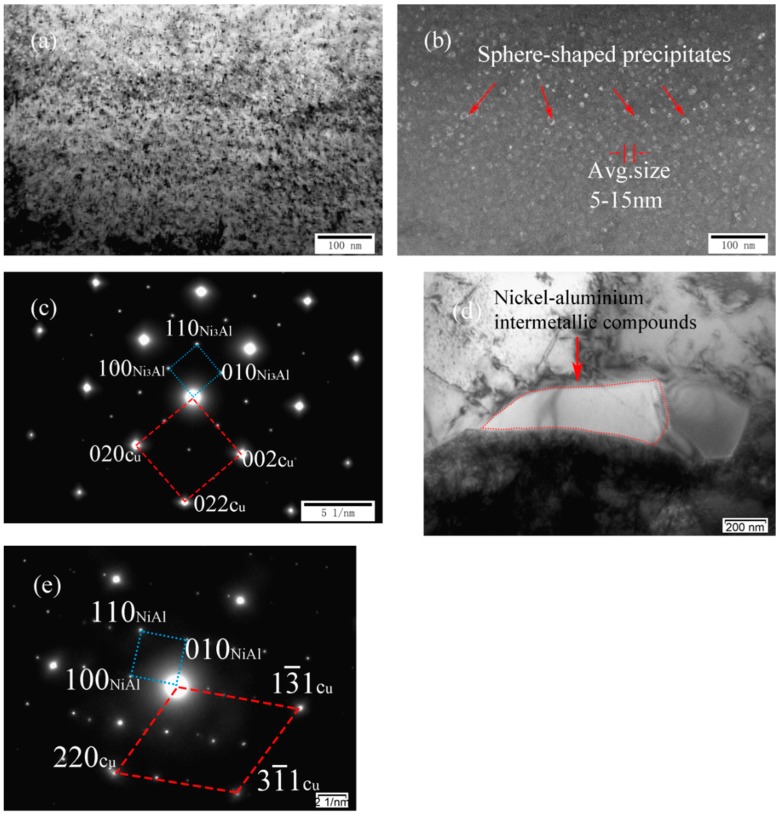
Microstructures of cast Cu-7Ni-5Al-1Fe-1Mn alloy: (**a**) bright-field image; (**b**) dark-field image; (**c**) the corresponding SADP of (**a**); (**d**) bright-field image in the grain boundary; and (**e**) the corresponding SADP of (**d**).

Figure 5a–d shows TEM images and SADP of as-cast Cu-7Ni-5Al-1Fe-1Mn alloy, respectively. As shown in Figure 5a,b, many sphere-shaped precipitates distribute on the copper substrate retained. Moreover, phase relationship between copper substrate and Ni_3_Al is found as follows (see Figure 5c): (002) Cu//(100)Ni_3_Al, (020) Cu//(010)Ni_3_Al, (022) Cu//(110)Ni_3_Al, [100] Cu//[001] Ni_3_Al. Particularly, a few irregular lumps in the grain boundary are also observed in Figure 5d. Analysis based on Figure 5e indicates the precipitates are one of the nickel–aluminum intermetallic compounds, namely NiAl, with a body centered cubic structure. Phase relationship between copper substrate and NiAl is found as follows: (1$\bar{3}$1) Cu//(100)NiAl, (3$\bar{1}$1) Cu//(110)NiAl, [1$\bar{1}$$\bar{4}$] Cu//[001] NiAl.

**Figure 6:**
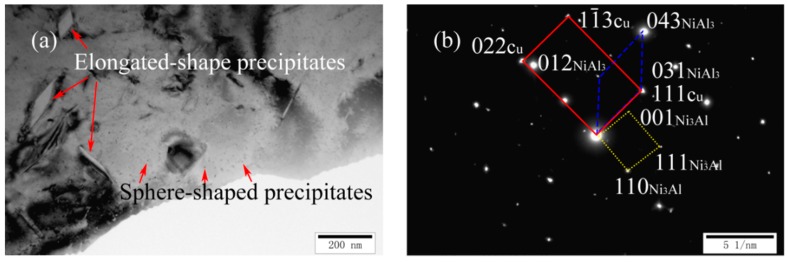
Microstructures of cast Cu-7Ni-7Al-1Fe-1Mn alloy: (**a**) bright-field image; and (**b**) the corresponding SADP of (**a**).

Figure 6a,b shows a TEM image and a corresponding SADP of as-cast Cu-7Ni-7Al-1Fe-1Mn alloy, respectively. Compared with cast Cu-7Ni-3Al-1Fe-1Mn alloy, there were significant changes in the size and morphology of precipitated phase. As observed in Figure 6a, the nano-scale precipitates still exist and the elongated precipitates appear at the same time. A precipitate having a width of several hundred nm is randomly distributed on the Cu-matrix. As seen from Figure 6b, the precipitates are one of the nickel–aluminum intermetallic compound,s namely NiAl_3_, with a orthorhombic structure. The crystal orientation relationship between the matrix and precipitate of Ni_3_Al and NiAl_3_ is: (022) Cu//(110) Ni_3_Al, (111) Cu//(031) NiAl_3_, [0$\bar{1}$1] Cu//[$\bar{1}$10] Ni_3_Al//[$\bar{5}$00] NiAl_3_.

As shown in Figure 2, Figure 3, Figure 4, Figure 5 and Figure 6, the precipitation behavior of Cu-7Ni-*x*Al alloy with the change of aluminum content has a series of phase transformation. The results show that, with the increase of aluminum content from 0% to 7%, the second-phase transformation of Cu-7Ni-*x*Al alloy follows the sequence of: α solid solution → Ni_3_Al → Ni_3_Al + NiAl → Ni_3_Al + NiAl_3._

### 3.2. Effect of Aluminum Content on Mechanical Properties

**Figure 7:**
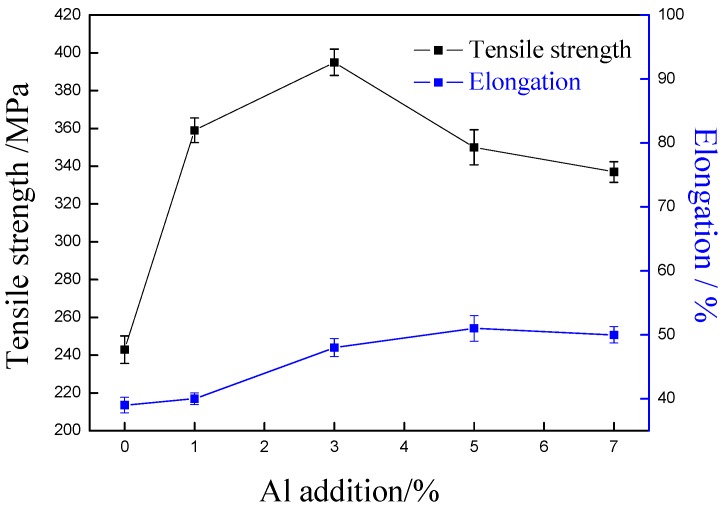
Effects of Al content on tensile strength and elongtion of Cu-7Ni-*x*Al-1Fe-1Mn alloy.

Figure 7 shows the tensile strength with different aluminum content. For comparison, aluminum has been found to be able to improve the mechanical strength of alloy. With the increase of aluminum content, the tensile strength of Cu-Ni-Al alloy firstly increases and then decreases, but all of them were higher than that of the copper alloy without aluminum content. The copper alloy with 3% aluminum has the highest tensile strength, 395 MPa. It was notable that, with the addition of aluminum content, the elongation of Cu-7Ni-*x*Al-1Fe-1Mn alloys firstly increases and then decreases, but still more than 40% higher than without aluminum content. The addition of aluminum element did not cause a severe impairment of tensile ductility.

As has been reported in Ref. [16,17], comparing copper with aluminum, the atomic radius and elastic modulus are quite different. After adding aluminum content, the elastic deformation degree of copper alloy matrix is very high. Aluminum is a very effective solid solution strengthener in copper alloy. The solid solubility of aluminum in Cu-Ni alloy is lower, and nickel–aluminum intermetallic compounds compound (Ni_3_Al, NiAl or NiAl_3_) are produced in Cu-Ni-A1 alloy (see Figure 3, Figure 4, Figure 5 and Figure 6), which has obvious precipitation hardening effect. From this, it is speculated that, in this study, solid solution hardening and precipitation hardening of aluminum are the reasons for enhancing the tensile strength. In particular, precipitation hardening is the main strengthening mechanism of Cu-Ni-Al alloys. When the aluminum content is 3%, the maximum tensile strength is the result of precipitation of a large amount of nano-Ni_3_Al. The experimental results have confirmed that the addition of aluminum significantly affects the mechanical properties of the Cu-Ni-Al alloy.

The plastic fracture of metal materials is the process of forming and growing microvoids. The fracture is the formation of microvoids in the place where the plastic deformation is serious. Under the action of tension, a large amount of plastic deformation makes brittle inclusions break or disconnect inclusions with the interface of the matrix to form holes [18]. Once the holes are formed, they begin to grow up and gather, and the result of the aggregation is the formation of cracks, which eventually lead to fracture. Smaller size and densely distributed Ni_3_Al particles promote dimple nucleation and form smaller and more dimple patterns. The size is larger and the irregular distribution NiAl_3_ particles promote crack growth and form larger dimple patterns. The change of the mechanical properties corresponds to the microstructure of the alloy. 

### 3.3. Effect of Aluminum Content on Corrosion Resistance

**Figure 8:**
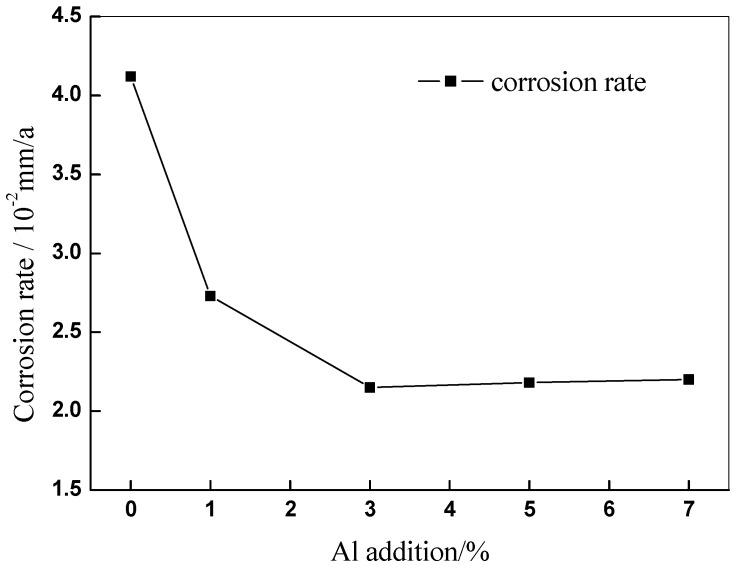
Effect of Al content on corrosion rate of Cu-7Ni-*x*Al-1Fe-1Mn alloy after immersing in artificial seawater for 10 days.

In Figure 8, the corrosion rate of Cu-7Ni-*x*Al-1Fe-1Mn alloy, after immersing simulated seawater for 10 days is plotted. The corrosion rate of copper alloy after addition of aluminum is lower than that of the copper alloy without aluminum. It can be determined that the addition of aluminum has a positive effect on the corrosion resistance of the alloy. With increasing aluminum content, corrosion rate firstly decreases and then has a slight increase. When the aluminum content is 3 wt %, the corrosion rate is the lowest, which is 0.0215 mm/a.

Such behavior has been explained in the literature on copper and copper alloys [19,20,21]. When immersed in chloride-containing solutions, the main anodic reaction of copper and its alloys is the dissolution process of copper according to

(2) $$\mathrm{Cu}+2{\mathrm{Cl}}^{-}\to \mathrm{Cu}{\mathrm{Cl}}_{2}^{-}+e^{-}$$

the cathode reaction is mainly the process of oxygen reduction

(3) $$O_{2}+2H_{2}O+4e\to 4{\mathrm{OH}}^{-}$$

According to H. Nady et al. [4], the copper chloride on the alloy surface will undergo further hydrolysis reaction, according to:

(4) $$2\mathrm{Cu}{\mathrm{Cl}}_{2}^{-}+H_{2}O\to {\mathrm{Cu}}_{2}O+4{\mathrm{Cl}}^{-}+2H^{+}$$

leading to the formation of cuprous oxide.

In the quinary, aluminum containing alloys can form the presence of Al-oxide, which can significantly reduce the chloride-induced corrosion compared with Cu metal, according to:

(5) $$\mathrm{Al}+4{\mathrm{Cl}}^{-}\to {\mathrm{AlCl}}_{4}^{-}+3e^{-}$$

(6) $${\mathrm{AlCl}}_{4}^{-}+2H_{2}O\to {\mathrm{Al}}_{2}O_{3}+3H^{+}+4{\mathrm{Cl}}^{-}$$

**Figure 9:**
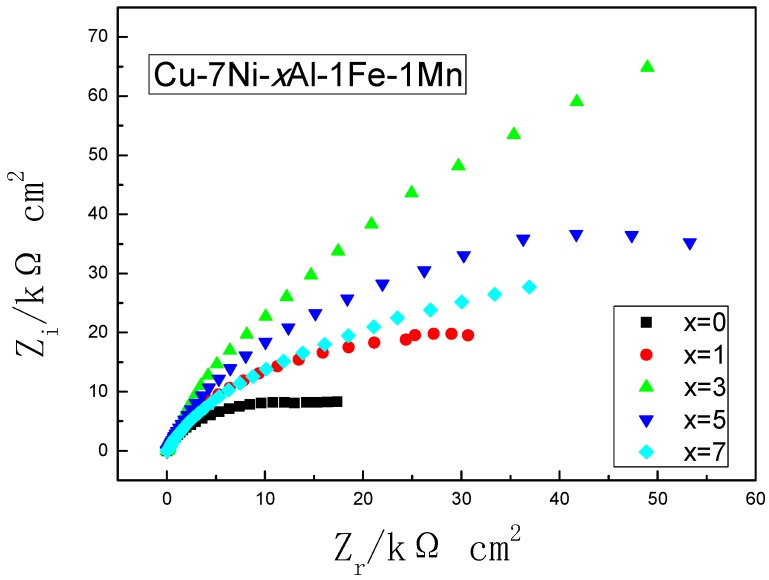
Nyquist impedance plots of the different Cu-7Ni-*x*Al-1Fe-1Mn alloy after immersing artificial seawater for 10 days.

Figure 9 is the impedance response of the different alloys in the artificial seawater after 10 days. The impedance spectrum shown in Figure 9 exhibits a similar characteristic, which is a single semicircular type. The overall trend of the diameter change of the Nyquist diagram is the gradual increase of the impedance Z, which means an increase in the transfer resistance of the oxide layer on the sample surface. The resistance of oxide film first increased and then decreased as the Al content increases which is in good agreement with the corrosion rate data.

The excellent corrosion resistance of copper alloy is attributes to the protective film formed on the surface. The addition of aluminum can form a compact oxide film [22] (mainly Cu, Ni and Al oxide film), which improves the quality of passive film and thus shields the alloy from corrosive ions. However, there is a certain potential difference between the precipitated phase and the substrate [9], which is not conducive to the improvement of corrosion resistance. Continuing to enhance the aluminum content in the alloy, the content of micron-scale metal compound distributes on the copper matrix, accelerating the alloy surface passivation film damage, which is unfavorable to the reduction of the corrosion rate of the alloy. These results are in agreement with the electrochemical results in Figure 9.

### 3.4. Mechanical Properties and Corrosion Resistance of Different Cupronickel

**Figure 10:**
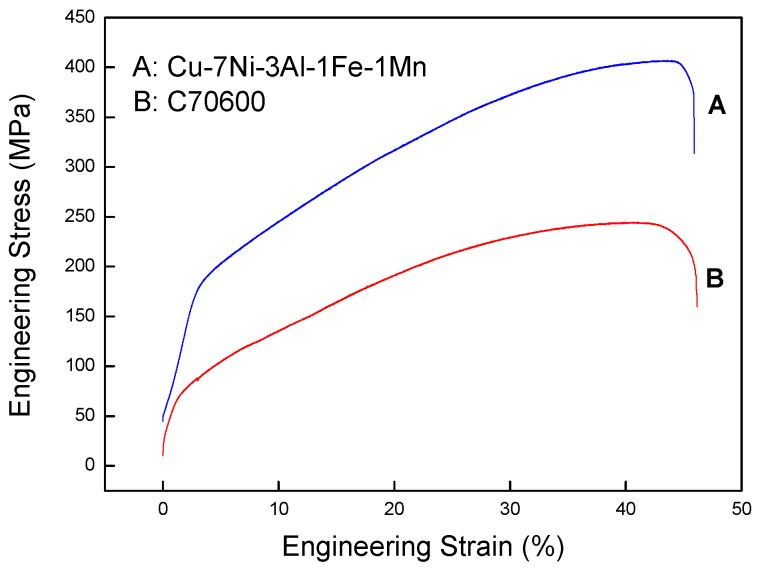
Engineering stress–strain curves at room temperature of the commercial alloy UNS C70600 and Cu-7Ni-3Al-1Fe-1Mn alloy.

The engineering stress–strain curves of the Cu-7Ni-3Al-1Fe-1Mn alloy and commercial alloy UNS C70600 are shown in Figure 10. To compare these alloys with each other directly, all data were obtained using the same method of the GB/T 2059-2008. Comparing the several Cupronickel alloys shows that the tensile strength becomes higher with increasing nickel content, with the designations of alloy B30-1-1 eutectic composition Cu-30Ni-1Fe-1Mn showing the best tensile strength. As shown in Figure 10, the tensile strength of Cu-7Ni-3Al-1Fe-1Mn is almost 61% higher than that of the commercial alloy UNS C70600 (Cu-10Ni-1Fe-1Mn). The addition of 3% aluminum has shown not only an improved tensile strength but also a much finer corrosion resistance, with the corrosion rate decreasing from 0.0359 mm/a to 0.0215 mm/a. Therefore, excellent corrosion resistance and good mechanical properties are expected for Cu-7Ni-3Al-1Fe-1Mn.

## 4. Conclusions

In this study, the effect of aluminum addition on mechanical properties and corrosion resistance in artificial seawater of Cu-7Ni-*x*Al-1Fe-1Mn alloy was investigated. Based on results obtained, the following conclusions can be drawn:

(1) The precipitation behavior of Cu-7Ni-*x*Al-1Fe-1Mn alloy with the change of aluminum content has a series of phase transformations. It is confirmed that the second-phase transformation of Cu-7Ni-*x*Al-1Fe-1Mn alloy follows the sequence of: α solid solution → Ni_3_Al → Ni_3_Al + NiAl → Ni_3_Al + NiAl_3_.
(2) The tensile strength of Cu-7Ni-*x*Al-1Fe-1Mn alloy first increased and then decreased but all of them were higher than that of the copper alloy without aluminum content. The Cu-7Ni-*x*Al-1Fe-1Mn alloy with 3wt % aluminum has the highest tensile strength with 395 MPa. Moreover, the addition of aluminum element did not cause a severe impairment of tensile ductility.
(3) Due to the formation of the stable Al_2_O_3_ in the barrier film, the aluminum can improve the stability of Cu-7Ni-*x*Al-1Fe-1Mn alloy. The corrosion resistance of the Cu-7Ni-*x*Al-1Fe-1Mn alloy in artificial seawater first decreased and then increased with the increase of aluminum content, but all of them were lower than that of the alloy without aluminum content.
(4) Among the Cu-7Ni-*x*Al-1Fe-1Mn alloys with aluminum content from 0% to 7 wt %, the alloy Cu-7Ni-3Al-1Fe-1Mn has the best mechanical properties and corrosion resistance in artificial seawater. Compared with commercial alloy UNS C70600, the tensile strength of Cu-7Ni-3Al-1Fe-1Mn alloy increased from 245 MPa to 395 MPa, and the corrosion rate decreased from 0.0359 mm/a to 0.0215 mm/a.

## Figures and Tables

**Table 1:** The nominal chemical compositions of the designed copper alloys (wt %).

Number	Cu	Ni	Fe	Mn	Al
1	Bal	7.0	1.0	1.0	0
2	Bal	7.0	1.0	1.0	1.0
3	Bal	7.0	1.0	1.0	3.0
4	Bal	7.0	1.0	1.0	5.0
5	Bal	7.0	1.0	1.0	7.0

**Table 2:** Artificial seawater composition.

Chemical Compound	Concentration, g/L	Chemical Compound	Concentration, g/L
NaCl	24.53	MgCl_2_	5.20
NaHCO_3_	0.201	CaCl_2_	1.16
KBr	0.101	KCl	0.695
H_3_BO_3_	0.027	Na_2_SO_4_	4.09
SrCl_2_	0.025	NaF	0.003
